# Minimizing recombinations in consensus networks for phylogeographic studies

**DOI:** 10.1186/1471-2105-10-S1-S72

**Published:** 2009-01-30

**Authors:** Laxmi Parida, Asif Javed, Marta Melé, Francesc Calafell, Jaume Bertranpetit, Genographic Consortium

**Affiliations:** 1 Computational Biology Center, IBM T J Watson Research, Yorktown, USA; 2 Department of Computer Science, Rensselaer Polytechnic Institute, New York, USA; 3 Biologia Evolutiva, Universitat Pompeu Fabra, Barcelona, Catalonia, Spain; 4 Work done during an internship at IBM T J Watson Research Center

## Abstract

**Background:**

We address the problem of studying recombinational variations in (human) populations. In this paper, our focus is on one computational aspect of the general task: Given two networks *G*_1 _and *G*_2_, with both mutation and recombination events, defined on overlapping sets of extant units the objective is to compute a consensus network *G*_3 _with minimum number of additional recombinations. We describe a polynomial time algorithm with a guarantee that the number of computed new recombination events is within *ϵ *= *sz*(*G*_1_, *G*_2_) (function *sz *is a well-behaved function of the sizes and topologies of *G*_1 _and *G*_2_) of the optimal *number *of recombinations. To date, this is the best known result for a network consensus problem.

**Results:**

Although the network consensus problem can be applied to a variety of domains, here we focus on structure of human populations. With our preliminary analysis on a segment of the human Chromosome X data we are able to infer ancient recombinations, population-specific recombinations and more, which also support the widely accepted 'Out of Africa' model. These results have been verified independently using traditional manual procedures. To the best of our knowledge, this is the first recombinations-based characterization of human populations.

**Conclusion:**

We show that our mathematical model identifies recombination spots in the individual haplotypes; the aggregate of these spots over a set of haplotypes defines a recombinational landscape that has enough signal to detect continental as well as population divide based on a short segment of Chromosome X. In particular, we are able to infer ancient recombinations, population-specific recombinations and more, which also support the widely accepted 'Out of Africa' model. The agreement with mutation-based analysis can be viewed as an indirect validation of our results and the model. Since the model in principle gives us more information embedded in the networks, in our future work, we plan to investigate more non-traditional questions via these structures computed by our methodology.

## Background

Reconstructing the recombinational history of a DNA fragment has proved to be a difficult problem and can only be achieved at small scales. Nonetheless the reconstruction of the history of long fragments, is of great interest to geneticists. Although the mutational history of adjacent fragments is independent, this is not true for recombinational history: thus merging adjoining networks add a new level of richness in complexity in terms of the suite of recombination events that shape variations within and across populations (both populations substructures as well as possible migratory history).

This paper explores the combinatorics involved in incorporating recombination events into the topology. While it is possible to give loose bounds on the number of recombination events using some convenient and clever variation of the *Four Gamete Rule *[1], the actual enumeration of the recombinations by a careful exploration of the underlying combinatorics will tighten this bound, as well as give additional information such as participating lineages, time-ordering of the recombination events and so on. However, it is important to note that the corresponding combinatorial optimization problem cannot be solved exactly unless P = NP [2,3]. Nevertheless, there have been various efforts to give a good estimate of a bound on this number (see [4] and citations therein).

In this paper, we address the problem of computing a consensus a pair of phylogenetic networks *G*_1 _and *G*_2 _to give *G*_3 _with a minimum number of new recombination events to jointly explain *G*_1 _and *G*_2_. Such a network *G*_3 _satisfies certain characteristics due to the very nature of its genesis: this is called a *compatible *network [5]. In this paper we presented a topology-based methodology to understand genetic variations in human haplotype data: We first cluster (possibly overlapping) haplotypes that display no evidence of recombinations and a representative haplotype of each cluster is extracted for the next phase. Then exploiting the coherence seen in such data, each haplotype is recoded using patterns of SNPs (patterns seen across different haplotypes). Finally, a network is constructed from the recoded representative haplotypes. Using a divide-and-conquer paradigm, the haplotype is segmented to give simple structures and then these individual structures are merged to give a unified topology using a DSR Scheme (see *Methods*). Clearly, each stage is algorithmically non-trivial, however optimizing the number of recombination events in the merging phase is a critical component. This is our focus in this paper. The interested reader is directed to [5] for other details including the rationale of the model.

In this paper, we analyze the performance of the DSR Scheme in two ways. Firstly, we give a mathematical evaluation of the algorithm. In other words, how far are we from the optimal number of new recombinations that explain the data? We show that the greedy polynomial time DSR based algorithm guarantees that the number of computed new recombination events is within *ϵ *= *sz*(*G*_1_, *G*_2_) (see Eqn 2) of the optimal number of recombinations. To date, this is the best known result for a network consensus problem. Note that the computation of consensus trees (or networks) is a very battered problem in literature. Thus, although our model is derived from the special setting discussed above, the problem and its solution is of interest in a general context involving reticulation events. See for example [6,7]. The ideas in pair-wise consensus is easily extendible to *k*-wise consensus.

Secondly, we examine how well the algorithm performs on real data. We apply the method on 100 Kb segment of high SNP density in the recombining part of the X chromosome. With our preliminary analysis from a phylogeographic viewpoint, we are able to infer ancient recombinations, population-specific recombinations and more (see *Experimental Results*) which also support the widely accepted 'Out of Africa' model. These results are consistent with established mutation-based methods: thus this can be taken as an indirect validation of our analysis and the methodology.

## Methods

Here we discuss the underlying mathematical model. We are given *H *units or extant individuals, each of which has *F *features. Each feature is a SNP (Single Nucleotide Polymorphism) and a unit is a haplotype. To keep the paper self-contained in this section we reproduce the notation used in [5]. A *network G *is a directed acyclic graph (DAG) and is defined as follows: It has three kinds of nodes. A node with no incoming edge is a *root *node and *G *may have multiple root nodes. A node with no outgoing edges is a *leaf *node. Each leaf node is labeled with nonempty sets haplotype labels. Every other node is an *internal *node. A node has at most two incoming edges. When a node has exactly one incoming edge, it is called a *mutation node *and the incoming edge is called a *mutation edge*. When the node has two incoming edges, the node is called a *recombination *or a *hybrid *or a *reticulation *node and the incoming edges are called *recombination *or *reticulation edges*. The direction of the edges is always towards the leaf nodes which in the figures in this paper is downwards. To avoid clutter, only the recombination edges display the direction as arrows.

Associated with a network *G *is a segmentation *S*. The *segmentation S *is a partition of the *F *features into some *k*(≤ *F*) (nonoverlapping) subsets. When the features are ordered say as *f*_1_, *f*_2_, *f*_3_,..., *f*_*F*_, they can be simply written as the closed interval [1, *F*], and the segmentation is a collection of non-overlapping intervals. For example, if *F *= 5, a possible segmentation of interval [1,5] is: *S *= {[1,2], [3,4], [5,5]}. The three individual segments are *s*_1 _= [1,2], *s*_2 _= [3,4] and *s*_3 _= [5,5] with *S *= {*s*_1_, *s*_2_, *s*_3_}. For convenience, the three segments are denoted simply by the consecutive integer labels 1, 2 and 3 and to keep clarity of exposition, *S *is written simply as *S *= {1, 2, 3}. Then each feature *f *is written as *s*: *f *where *s *is the segment label that the feature *f *belongs to.

**Figure 1:**
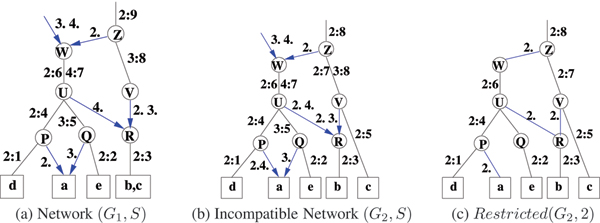
In (a) & (b) *G*_1 _and *G*_2 _have segmentation *S *= {2, 3, 4}. (b) The two parents of node 'R' have labels {4, 2} and {3, 2}. Thus, the network restricted to segment label 2, shown in (c), has a closed path defined by the nodes labeled 'Z', 'W', 'U', 'R' and 'V'. Hence the network in (b) is not compatible.

For a segmentation *S*, the labeling of the edges of *G *are as follows: (1) Mutation edge: Every mutation edge *e *incident on a node *v*, has a non-empty label, *lbl*. Each member, *s*: *f*, of the label is interpreted as feature *f *in segment *s*. (Note that *f *itslf may have the form '2:3' as in Figure 1. Now, if *f *is associated with segment 9, the label is written as 9:2:3.) Further, each element appears at most once in an edge label in *G*. (2) Recombination edge: The two recombination edges, *e*_1 _and *e*_2_, incident on a recombination node *v *are labeled by sets of segment labels *lbl*_1 _and *lbl*_2 _with *lbl*_1_≠ *lbl*_2_. For example *lbl*_1 _= {1, 3} denoting that *e*_1 _is labeled with the segment labels 1 and 3.

For a segment *s *∈ *S*, *Restricted*(*G*, *s*) is the network obtained by doing the following two operations:(1) Removing all recombination edges in *G *that do not have the element *s *in the true edge label *lbl'*. (2) From each mutation edge label in *G*, removing the elements of the form *s*: *f*, for any *f*, from the edge label. For a concrete example see Figures 1(b) and 1(c). This definition is easily extended to multiple segments as *Restricted*(*G*, *S'*), where *S' *⊆ *S*.

*G *is always associated with a segmentation *S *of the *F *features, hence written as (*G*, *S*). Note that *G *cannot be any arbitrary network. It must satisfy the following: for each *s *∈ *S*, *Restricted*(*G*, *s*) is devoid of recombinations. Such a network is termed *compatible*. The *Consensus Compatible Network Problem *is defined as follows [5]: Given two compatible networks (*G*_1_, *S*_1_) and (*G*_2_, *S*_2_) with no common features (thus *S*_1 _∩ *S*_2 _= ∅), the task is to compute a compatible network (*G*_3_, *S*_1_∪ *S*_2_) with the minimum number of additional recombination nodes such that (*G*_1_, *S*_1_) is isomorphic to *Restricted*(*G*_3_, *S*_1_) and (*G*_2_, *S*_2_) to *Restricted*(*G*_3_, *S*_2_).

In the remainder of the paper, we refer to (*G*, *S*) simply as *G*, and segmentation *S *will be clear from context.

### The Dominant Subdominant Recombinant (DSR) framework

The DSR scheme to solve the problem and its proof of correctness was presented in [5]. The method is an iterative, bottom-up working at one *level *of *G*_1 _and *G*_2 _at a time. The level of a node is defined as the maximum distance to a leafnode.

The same level is also associated with any edge *e *incident on the node written as *level*(*e, G*). A leaf is at level 0. The method gets its name from the need to give one of three possible assignments, Dominant (D) or Subdominant (S) or Recombinant (R), to nodes at each iteration, which is central to this scheme.

### Matrix *X*_*l*_

**Figure 2:**
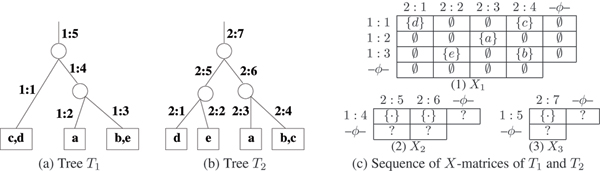
Given trees *T*_1 _in (a) and *T*_2 _in (b), each of height 3. (c) These two trees define *X*_*l*_, 1 ≤ *l *≤ 3, for each level *l*. Note that the entries in *X*_*l*_, *l *> 1 differ in details depending on the choices the DSR algorithm makes. While '∅' denotes an empty set, '?' (including '{·}') could be either empty or non-empty, again depending on the choices the DSR Scheme makes.

Let *G *have *t *roots. For root *v*_*i *_introduce an incoming edge *e*_*i*_, 1 ≤ *i *≤ *t*. Then the *height *of *G *is defined as $\max_{i=1}^{t}$ (*level*(*e*_*i*_, *G*)). Let *l*_max _(*l*_min_) be the maximum (minimum) of the heights of *G*_1 _and *G*_2_. For a fixed level *l*, let (*i *= 1, 2), $E_{i}^{l}$ = {*e *is an edge in *G*_*i *_| *level*(*e*, *G*_*i*_) = *l*}. Then intersection *n*_*l *_× *m*_*l *_matrix *X*_*l *_is defined as $X_{l}=E_{1}^{l}\times E_{2}^{l}$ and an example is shown in Fig 2. In the algorithm the intersection matrix, *X*_*l *_had dimensions (*n*_*l *_+ 1) × (*m*_*l *_+ 1) as this extra last row (column) with header '-*ϕ*-' is required to take care of elements that are not covered by the rest of the columns (or rows). An empty entry is shown as '∅'. In *X*_1 _the exact entries can be computed and for *X*_*l*_, *l *> 1 and the non-empty entries are identified by '{·}'. Further, let *x*_*l *_be the number of non-empty entries in *X*_*l*_. See Figure 2 for an example.

### DSR assignment rules

**Figure 3:**
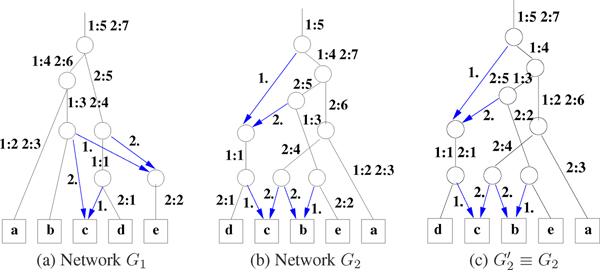
(a) & (b) Two possible consensus networks *G*_1 _and *G*_2 _for two input trees *T*_1 _and *T*_2 _of Figure 2. (c) The edge labels of *G*_2 _have been locally shuffled keeping the exact same topology.

**Figure 4:**
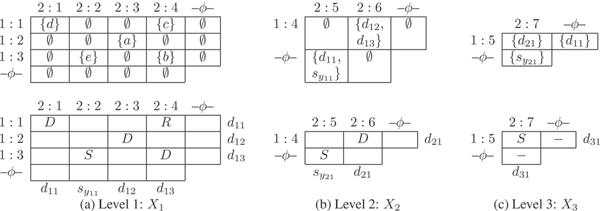
*X*-matrices of Network *G*_1 _of Figure 3(a). The *X*_*l *_matrix is shown on the top and the DSR assignment shown in the bottom row for each *l*, 1 ≤ *l *≤ 3.

**Figure 5:**
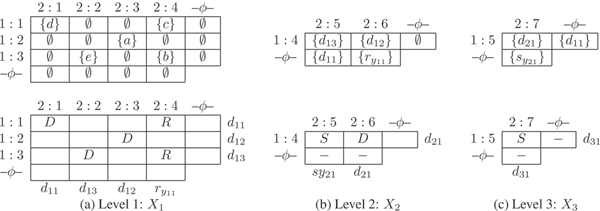
*X*-matrices of Network *G*_2 _of Figure 3(b). Also see Figure 4 for a description of the matrices.

**Figure 6:**
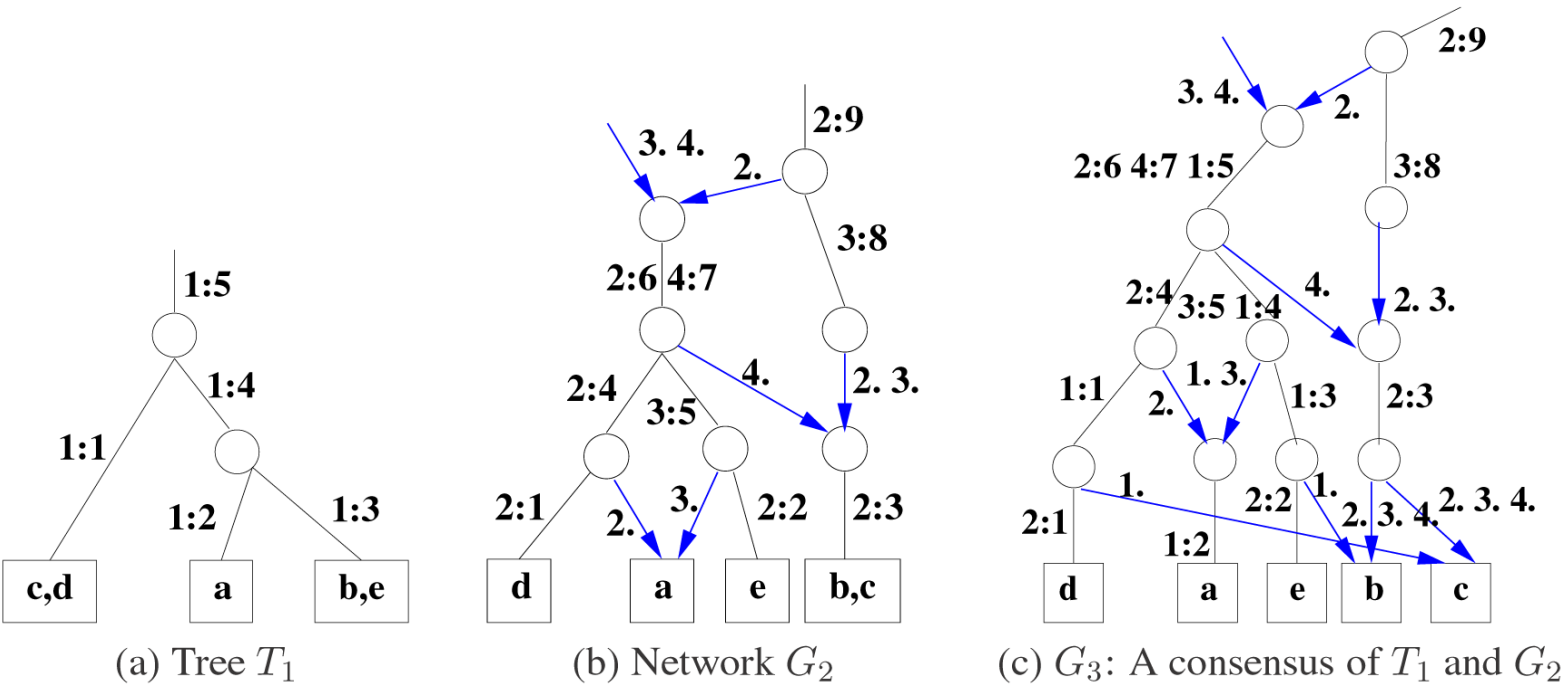
Consensus of a tree and a network.

**Figure 7:**
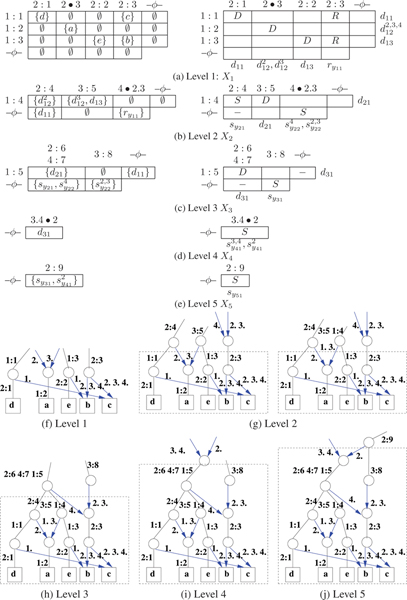
Stepwise construction of *G*_3 _of Figure 6(c) as consensus of *T*_1 _and *G*_2_: (a)–(e) The *X *matrices and the DSR assignments. (f)–(j) The construction of *G*_3 _using the DSR assignments of (a)–(e).

The non-empty entries of *X*_*l *_are given a DSR assignment. Note that at least two conditions are required for a viable compatible network *G*_3_. (Rule 1): Each row (column) in matrix *X*_*l *_has at most one dominant. If the row (column) has no dominant, then it has at most one subdominant. (Rule 2): A non-recombinant element can have another non-recombinant in its row or its column but not both. As a result of the DSR assignments to the entries on *X*_*l*_, the rows and columns also get implicitly assigned as follows. A row (column) that has a dominant entry is assigned dominant. A row (column) that is not assigned dominant but has a subdominant in the row (column) gets assigned subdominant. A row (column) that has only recombinants in the row (column) is assigned recombinant. Note that only dominant rows (columns) contribute to entries in *X*_*l*'_, *l' *> *l*. Figures 4 and 5 give two different assignments giving the two different networks in Figure 3 (a) and (b) respectively. Using a simple *greedy *optimization approach, we include a third rule. (Rule 3): Minimize the number of recombinants in *X*_*l*_. Complete examples are worked out in Figures 4, 5, 6 and 7 for the interested reader.

### Approximation factor of the greedy DSR scheme

In this section, we compute the approximation factor [8,9] of the greedy version of the DSR Scheme. Let the number of new recombination events produced by the DSR algorithm in *G*_3 _be *N*_DSR_. Let the optimal number of new recombinations be *N*_opt_. We use the following definition of the true approximation factor:

(1) $$approx_{true}=\frac{N_{\text{DSR}}-N_{\text{opt}}}{N_{\text{opt}}}.$$

For given graphs *G*_1 _and *G*_2 _let *z*_*l *_= max(*n*_*l*_, *m*_*l*_) where *n*_*l *_> 0 and *m*_*l *_> 0 are the number of nodes at level *l *in *G*_1 _and *G*_2 _respectively. Further, let *Z *be the sum of all *z*_*l *_over all the levels (excluding the leaf level). Let *L*_*v*_(*G*) be all the leafnodes (extant units) reachable from node *v *in *G*. For each level, *l *> 0, i.e. excluding the leafnodes, consider $L_{v_{i}}$ (*G*_1_), 1 ≤ *i *≤ *n*_*l*_, where each *v*_*i *_is at level *l *in *G*_1_. Similarly consider $L_{u_{i}}$ (*G*_2_), 1 ≤ *i *≤ *m*_*l*_, where each *u*_*i *_is at level *l *in *G*_2_. Let *x*_*l *_be the number of non-empty intersections between the two collection of sets and let *Y *be the sum of *x*_*l *_over all the levels (excluding leaf level). Note that if *G*_1 _and *G*_2 _are the same (isomorphic) graphs then *Y *= *Z *and *N*_opt _= 0.

### Theorem 1

(2) $$approx_{true}\leq \frac{Z}{\max(1,Y-Z)}.$$

*Proof: *Let *N*_max _(*N*_min_) be the maximum (minimum) number of new recombinations produced by the DSR scheme over all possible DSR assignments. Then we first show the following:

(3) *N*_min_≤ *N*_opt_≤ *N*_DSR_≤ *N*_max_.

Clearly *N*_opt_≤ *N*_max _holds (else it contradicts the optimality of *N*_opt_). Next we have to show that *N*_min _≤ *N*_opt _holds as well. For this we need a few more characterizations of the network.

### Recombination node descriptor *F*_1_|*F*_2_

Let *Y *be the set all given haplotypes (or taxa). A *split *or *bipartition *is written as *Z*_1_|*Z*_2 _where *Z*_1 _and *Z*_2 _are nonoverlapping subsets of *Y *with *Y *= *Z*_1_∪ *Z*_2_. A *tripartition Z*_1_|*Z*_2_|*Z*_3 _is defined similarly. In earlier works (see [7,2] and citations therein) a mutation event has been associated with a bipartition of *Y *and a recombination event with a tripartition. However, the latter requires certain restrictions in the form of network *G*, i.e., a recombination node cannot be a direct descendent of another recombination node. Here we define recombination nodes as a bipartition of an appropriate subset of features.

For a fixed segment *s*, let *s*-path be a path in the graph with mutation edge(s) and recombinant edge(s) with *s *in its label. For any *v*, note that there is a unique *s*-path from a root to *v*. Further, let *v *be a recombination node and *lbl*_1 _and *lbl*_2 _be the labels of the two incoming (recombination) edges *u*_1_*v *and *u*_2_*v *respectively. For *s*_1 _∈ *lbl*_1 _but *s*_1 _∉ *lbl*_2_, let feature *f*_1 _be such that *s*_1 _: *f*_1 _is in the label of the closest mutation edge on the *s*_1_-path from *v*. Then *F*_1 _is the set of all such features. *F*_2 _is defined similarly. For example in *G*_1 _of Figure 1(a), consider the recombination leafnode labeled with haplotype *a*. Here *lbl*_1 _= {2}, *lbl*_2 _= {3} and the descriptor for this node is *F*_1_|*F*_2 _= {2:4}|{3:5}. For the recombination node labeled 'R', *lbl*_1 _= {4}, *lbl*_2 _= {2, 3} and the descriptor is *F*_1_|*F*_2 _= {4:7} | {2:9, 3:8}.

### Isomorphism (*G*_1 _≡ *G*_2_))

Let *L*_*v*_(*G*) be all the leafnodes (extant units) reachable from node *v*. Let *s*: *f *be in the label of the unique incoming edge on mutation node *v *and then let *L*_*s*: *f *_(*G*) be the same as *L*_*v*_. Two compatible networks *G*_1 _and *G*_2 _on the same segmentation *S *are *isomorphic *(or identical), written as *G*_1 _≡ *G*_2_, if the following two conditions hold: (1) For each element *s*: *f *in *G*_1_, *L*_*s*: *f *_(*G*_1_) = *L*_*s*: *f *_(*G*_2_) and viceversa, and, (2) For each recombination node *v *in *G*_1 _with descriptor *F*_1_|*F*_2_, there exists a recombination node in *G*_2 _with the same descriptor and viceversa.

### Canonical form

It is possible to bubble *up *or *down *an element in the mutation edge label to obtain *G' *such that *G' *≡ *G*. Our convention will be to bubble *down *the element of the mutation edge label, towards a leafnode. A network *G *is in the *canonical form *(1) if no node has only one outgoing edge and (2) if no element of any mutation edge label can be bubbled down to obtain *G' *with *G' *≡ *G*. For example see Figure 3. Since the levels of nodes in a canonical network are unique, the following can be readily verified (see also concrete examples in Figures 2 and 6).

**Lemma 1 ***Let G*_3 _*be the consensus of G*_1 _*and G*_2 _*which are in canonical forms, with l*_max _*(l*_min_*) as the maximum (minimum) of the heights of G*_1 _*and G*_2_. *Then there exist some X-matrices, X*_1_, *X*_2_,..., $X_{l_{\max}}$*whose DSR assignments produce G*_3_. *This is written as G*_3 _≅* X*_1_, *X*_2_, ...,$X_{l_{\max}}$.

*Back to the proof: *We have to show that *N*_min _≤ *N*_opt _holds. Assume the contrary, i.e., *N*_opt _<*N*_min_. In other words, the optimal number of new recombinations is even lower than the minimum produced by the algorithm over all possible choices. Then consider this network ${G^{\prime }}_{3}$ with *N*_opt _new recombinations. Then by Lemma 1, there exist a sequence of *X*-matrices ${G^{\prime }}_{3}$ ≅ *X*_1_, *X*_2_, ..., $X_{l_{\max}}$ with some DSR assignments for each *X*_*l*_. Thus by these choices of the algorithm *N*_min _≤ *N*_opt _must hold, again leading to a contradiction.

Hence *N*_opt _≮ *N*_min_. Here ends the proof of correctness of Eqn 3. Next, we give a few characterizations of

the DSR assignment to facilitate the counting of the new recombinations.

### Type I & II (new) recombination events

Let *v *be a recombination node in *G*_3 _with labels *lbl*_1 _and *lbl*_2 _on the two incoming edges and descriptor *F*_1_|*F*_2_. The recombination event is *new *if, without loss of generality, *lbl*_1_⊆ *S*_1 _and *lbl*_2_⊆ *S*_2_. In other words, this recombination node is a result of the consensus of *G*_1 _and *G*_2 _(and not a recombination that existed in *G*_1 _or *G*_2_). A new recombination node *v *is of two types: Let *e*_1 _(*e*_2_) be a mutation edge in *G*_1 _(*G*_2_) with a label in *F*_1 _(*F*_2_). Without loss of generality, let *level*(*e*_1_, *G*_1_) = *l*. Then the recombination is of Type I at level *l *if *level*(*e*_2_, *G*_2_) = *l *and is of Type II at level *l *if *level*(*e*_2_, *G*_2_) > *l*. Further, let the number of (non-empty) entries assigned dominant be $n_{l}^{D}$, subdominant be $n_{l}^{S}$ and recombinant be $n_{l}^{R}$ in an *X*-matrix *X*_*l*_. Then the following can be verified.

**Lemma 2 ***The number of Type I recombination events at level l in G*_3 _*is *$n_{l}^{R}$. *The number of Type II recombination events at level l in G*_3 _*is *≤ $n_{l}^{D}+n_{l}^{S}$. *Also, the number of recombination events in a network is bounded below (N*_min_*) by the number of Type I recombination events and above (N*_max_*) by the sum of the number of Type I and Type II recombination events*.

### Islands in *X*

**Figure 8:**
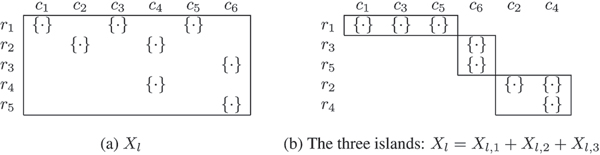
(a) *X*_*l *_has five rows and six columns. (b) The rows and columns have been permuted (shuffled) to reveal the three islands (or three connected components in the associated bipartite graph).

We now give tighter bounds on $n_{l}^{D}$, $n_{l}^{S}$ and $n_{l}^{R}$ for our analysis. Consider a bipartite graph *B*(*V, E*) with *V *partitioned into (1) *n*_*l *_nodes, corresponding to the rows and (2) *m*_*l *_nodes corresponding to the columns of *X*_*l*_. The adjacency matrix ${X^{\prime }}_{l}$ is obtained from *X*_*l *_where an empty set entry is replaced with 0 and a non-empty set entry with 1. Let the number of connected components [10] of graph *B*(*V, E*) be *C*_*l*_. Each connected component corresponds to an *island *in *X*_*l *_which is a collection of rows and columns of *X*_*l*_. Thus *X*_*l *_is fragmented into *C*_*l *_islands, *X*_*l*,*i*_, written as: *X*_*l *_= *X*_*l*,1 _+ *X*_*l*,2 _+ ... + $X_{l,C_{l}}$. See Figure 8 for an example. Note that this fragmentation is for analysis purposes only. Further, $\sum_{l=1}^{l_{bnd}}\sum_{i=1}^{C_{l}}y_{l,i}$, for any *y*_*l*,*i*_, will be written simply as $\sum_{l,i}^{l_{bnd}}y_{l,i}$. Let island *X*_*l*,*i *_have *x*_*l*,*i *_non-empty entries and let the number of entries assigned *Y *(*D *or *S *or *R*) in *X*_*l*,*i *_be $n_{l,i}^{Y}$. Within an island the number of non-recombinants cannot exceed max(*n*_*l*,*i*_, *m*_*l*,*i*_) by Rules 1 and 2.

**Lemma 3 ***For each island X*_*l*,*i*_:

(4) $$n_{l,i}^{D}+n_{l,i}^{S}=\max(n_{l,i},m_{l,i}),$$

(5) $$n_{l,i}^{R}=x_{l,i}-\max(n_{l,i},m_{l,i}).$$

Eqn 4 follows from using Rule 3 in island *X*_*l*,*i *_and Eqn 5 from $x_{l,i}=n_{l,i}^{D}+n_{l,i}^{S}+n_{l,i}^{R}$.

**Figure 9:**
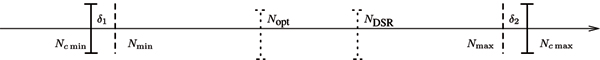
The relative positions of the different counts on the real line. See text for details.

*Back to the proof: *Next, let *N*_*c *max_(≥ *N*_max_) and *N*_*c *min_(≤ *N*_min_) be some computable functions of the input (see Figure 9). Using Lemmas 2 and 3, we define appropriate (computable) *N*_*c *max_and *N*_*c *min_as follows:

(6) $$N_{\max}\leq \sum_{l}^{l_{\min}}x_{l}=N_{c\max}$$

(7) $$\begin{array}{c} N_{\min}=\sum_{l,i}^{l_{\min}}n_{l,i}^{R}=\sum_{l,i}^{l_{\min}}\left(x_{l,i}-\max(n_{l,i},m_{l,i})\right)\\ \geq \sum_{l}^{l_{\min}}x_{l}-\sum_{l}^{l_{\min}}\max(n_{l},m_{l})=N_{c\min} \end{array}$$

Note that the greedy Rule 3 encourages fragmentation of *X*_*l*_, *l *> 1, into islands and under the best case scenario we get $n_{l}^{D}+n_{l}^{s}=\sum_{l}^{l_{\min}}\max(n_{l},m_{l})$, which is used in Eqn 7 above. Finally, using Eqn 1, we have

(8) $$\begin{array}{c} approx_{true}=\frac{N_{\text{DSR}}-N_{\text{opt}}}{N_{\text{opt}}}\leq \frac{N_{c\max}-N_{c\min}}{N_{c\min}}\\ \approx \frac{N_{c\max}-N_{c\min}}{\max(1,N_{c\min})} \end{array}$$

The correctness of Eqn 2 is established by setting $Z=\sum_{l,i}^{l_{\min}}\max(n_{l},m_{l})$ and $Y=\sum_{l}^{l_{\min}}x_{l}$. Here ends the proof.

## Experimental results and discussion

In the last section we gave a mathematical proof of the tightness of the number of recombinations estimated by the model to explain the data. Also, in our earlier work we had presented results on simulation data with a general analysis of false positive and false negative errors. In this section, we discuss results on a segment of Chromosome X data and the plausibility of the results is verified independently by using traditional manual analysis. Due to the manual component in the verification process, here we use only small data sets.

### Chromosome X SNP data

We used a 100 Kb segment of high SNP density in the recombining part of the X chromosome, starting at genomic position 87,348,404 (Build 35). In Hapmap II [11], this segment contains 194 sites, of which only 175 are polymorphic in at least one population. Recombination rate is ≈ 0.7 cM/Mb, slightly below the ≈ 1 cM/Mb genomewide average. We chose this segment for two reasons. (1) It does not contain any genes. Thus variation in this region is less likely to have been shaped by natural selection and is more likely to reflect purely genomic processes. (2) The segment does not contain copy number variations or segmental duplications. These could induce genotyping errors possibly producing ectopic recombination events, which is not accounted for in the downstream analysis.

Further, we used only the haplotypes in the hemizygous males to avoid any phasing errors. These errors would manifest as phantom recombination events. The populations used were Yorubans from Nigeria (YRI; *N *= 30), Europeans (CEU, *N *= 30), and a pooled sample of Chinese and Japanese (ASN; *N *= 45).

### Statistical analysis (using *p*-value estimations)

As a preprocessing step, exploiting the coherence seen in the data, each haplotype is recoded using blocks of *g *SNPs. Based on the combinatorial model, a network is constructed from the recoded representative haplotypes. Recall that first the haplotype is segmented to give simple structures and then these individual structures are merged with a small number of recombinations to give a unified topology. Here we discuss the choice of the value of *g *in our experiments. Let *l *be the number of distinct patterns of the *g *SNPs across the samples. Using this as a proxy for the extent of LD in this block, we estimate the *p*-value of the number *l*. Loosely speaking, when these *g *SNPs are in linkage equilibrium (or independent), *l *should be much larger than when they are in LD.

Let the number of samples be *H *and let the number of SNPs be *F*. Further, let *V *be a column vector of size *H*. Since the SNPs are assumed to be bi-allelic, *V *which represents the value of a SNP in the *H *samples is binary. We use two schemes, based on the mode of definition of the *F *vectors, to estimates the *p*-value. The range of values of *l *seen in our data is 2 ≤ *l *< 15 and we study the *p*-value estimates in this range using two schemes.

#### RandV

**Figure 10:**
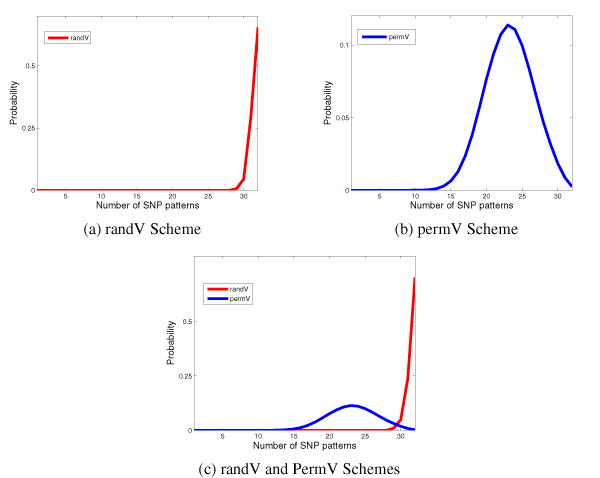
Distribution of *l *for *g *= 5. Recall that the randV Scheme is independent of the region but the permV Scheme uses the population distribution of the region for a more realistic estimation.

In this scheme, *V*_1_, *V*_2_, ..., *V*_*F *_are defined randomly. In other words, each entry of each *V *is picked independently and uniformly from a set of two alleles. We use 10000 replicates and the distribution of the number of *g*-sized patterns is shown in Fig 10. The *p*-values estimated based on this scheme is shown in the table below. The *p*-values are ≈ 0.0 for every value of *l*.

#### PermV

While the RandV scheme is not incorrect, we make some domain-dependent modifications to design another scheme. In the PermV scheme we (i) mimic the allele frequencies seen in the input data and (ii) use the population distribution, by ethnicity, of the screened samples in the chromosomal region. The individual *V *vectors are plucked from the *X*-Chromosome of the database (but the SNPs span the entire chromosome) and any untyped SNP (i.e., N in the database) in the vector is given a value in agreement with the allele frequency of that column. Further, we use only those *V *'s that have MAF ≥ 0.1. We again use 10000 replicates and for each replicate, we randomly permute the *F *vectors. The distribution of the number of *g*-sized patterns is shown in Fig 10(b).

If for a block, *l *has an insignificant *p*-value, then the subsequent analysis risks becoming unreliable. We then reduce the grain size. An alternative is to discard the offending SNPs of the block, thus fragmenting the region. In our experiments we used a grain size *g *= 5 and the *p*-values obtained for this on all the regions were acceptable. The haplotypes are re-coded as sequence of these SNP patterns for the combinatorial analysis discussed in the *Methods *section.

### Result analysis

**Figure 11:**
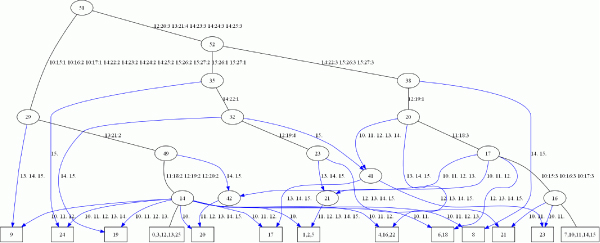
The network on segment Chr X: 87390235–87412114 of the three populations. The leafnodes are labeled with (a set of) clusters of the input haplotypes. A label on an internal node is for reference purposes only. An element of the edge label is to be interpreted as segment-id:position-id:pattern-id.

We show a sample network of a short segment of the chosen region in Figure 11. Here we summarize our observations from a phylogeographic viewpoint and answer only questions raised traditionally in this area. Table 1 shows the number of detected recombination events and how they are shared across populations. The observations (over the entire 100 Kb segment) are as follows: We discovered a total of 31 recombinations in the data. Seventeen recombinations are population-specific, and can be used to infer the recombinational diversity within a population. Assuming recombination rate is constant across populations, this is a function of the effective population size of each population. Four recombinations are shared among pairs of populations, and can be used as indicators of shared population ancestry. In this particular case, both Europeans and Asians share events with the African population, which is more recombinationally diverse. Ten recombinations are shared among all three populations, and they are presumably ancient events that occurred before the split of the three populations.

**Table 1:** 

	CEU	ASN	YRI
CEU	2	0	1
ASN		4	3
YRI			11

CEU & ASN & YRI: 10

Mutation-based studies of genetic diversity have shown exactly the same pattern: a larger diversity in Africans, and variation outside of Africa that is partially a subset of that in Africa. Our recombination-based results follow the same pattern, and, as the mutation data, fit the the "Out of Africa" model [12] for the origin of anatomically modern humans. Consistency with mutation data can be taken as an indirect validation of our analysis and the methodology. In our future work, we plan to investigate (raise as well as answer) more non-traditional questions.

## Conclusion

We have addressed the problem of studying recombinational variations in human populations. One of the contributions of this work is a guaranteed upper bound on the approximation factor (ratio of discovered new recombination events to the true optimal) in a greedy polynomial time algorithm to construct a consensus network. Such an assurance is of significance when dealing with data where there are no other reasonable means of cross-checking results. To date, this bound is the best known result for this problem. We use this scheme to study recombinational imprints in an appropriate segment of X chromosome from three populations. While the upper bound on the approximation is our theoretical contribution, our second contribution is the results on this data: With our preliminary analysis, we are able to infer ancient recombinations, population-specific recombinations and more, which also support the widely accepted 'Out of Africa' model. The agreement with mutation-based analysis can be viewed as an indirect validation of our results and the methodology. In our future work, we plan to investigate more non-traditional questions via the networks computed by our methodology.

## Competing interests

The authors declare that they have no competing interests.

## Authors' contributions

The work is a result of the synergistic efforts of all the authors. However, the brunt of each author's involvement is as follows. Design and analysis of the mathematical models: LP. Design and implementation of the algorithms: AJ. Design and implementation of the experiments: MM, FC and JB. Further, LP and JB were involved in conceiving and planning the recombinations project.
